# Optimization for the Production of Deoxynivalenol and Zearalenone by *Fusarium graminearum* Using Response Surface Methodology

**DOI:** 10.3390/toxins9020057

**Published:** 2017-02-10

**Authors:** Li Wu, Lijuan Qiu, Huijie Zhang, Juan Sun, Xuexu Hu, Bujun Wang

**Affiliations:** 1 Institute of Crop Science, Chinese Academy of Agricultural Sciences, No.12 Zhongguancun South St., Haidian District, Beijing 100081, China; wuli5151@126.com (L.W.); qiulijuan@caas.cn (L.Q.); zhanghuijie01@caas.cn (H.Z.); gwzx848@163.com (J.S.); huxuexu@caas.cn (X.H.); 2 Laboratory of Quality and Safety Risk Assessment for Cereal Products (Beijing), Ministry of Agriculture, Beijing 100081, China

**Keywords:** *Fusarium graminearum*, deoxynivalenol, zearalenone, response surface methodology, optimization, purification

## Abstract

*Fusarium* mycotoxins deoxynivalenol (DON) and zearalenone (ZEN) are the most common contaminants in cereals worldwide, causing a wide range of adverse health effects on animals and humans. Many environmental factors can affect the production of these mycotoxins. Here, we have used response surface methodology (RSM) to optimize the *Fusarium graminearum* strain 29 culture conditions for maximal toxin production. Three factors, medium pH, incubation temperature and time, were optimized using a Box-Behnken design (BBD). The optimized conditions for DON production were pH 4.91 and an incubation temperature of 23.75 °C for 28 days, while maximal ZEN production required pH 9.00 and an incubation temperature of 15.05 °C for 28 days. The maximum levels of DON and ZEN production were 2811.17 ng/mL and 23789.70 ng/mL, respectively. Considering the total level of DON and ZEN, desirable yields of the mycotoxins were still obtained with medium pH of 6.86, an incubation temperature of 17.76 °C and a time of 28 days. The corresponding experimental values, from the validation experiments, fitted well with these predictions. This suggests that RSM could be used to optimize *Fusarium* mycotoxin levels, which are further purified for use as potential mycotoxin standards. Furthermore, it shows that acidic pH is a determinant for DON production, while an alkaline environment and lower temperature (approximately 15 °C) are favorable for ZEN accumulation. After extraction, separation and purification processes, the isolated mycotoxins were obtained through a simple purification process, with desirable yields, and acceptable purity. The mycotoxins could be used as potential analytical standards or chemical reagents for routine analysis.

## 1. Introduction

Fungi in the genus *Fusarium* are important pathogens of small-grain cereals, including wheat, maize, barley, and oats, especially in the temperate regions of the world [1,2]. Toxigenic fungi produce a variety of toxic metabolites that contaminate cereal grains and cereal-based food products, resulting in economic losses and potentially threatening the health of humans and animals [3,4,5]. *Fusarium* head blight (FHB), a common fungal disease of cereals, is caused by several *Fusarium* species worldwide, including *F. graminearum*, *F. asiaticum*, *F. avenaceum*, *F. culmorum*, *F. tricinctum*, *F. langsethiae*, *F. sporotrichioides* and *F. poae* [6,7,8]. Among these species, *F. graminearum* is ubiquitous and the most prevalent species in temperate regions, such as China, United States and other countries [9,10]. However, *F. asiaticum* is also a dominant species in China, especially in the southern rainy regions [11].

*F. graminearum* produces zearalenone (ZEN) and trichothecene mycotoxins, such as deoxynivalenol (DON), 3-acetyl-deoxynivalenol (3-ADON), 15-acetyl-deoxynivalenol (15-ADON), nivalenol (NIV) and 4-acetylnivalenol (4-ANIV) [12,13]. Based on the different type B trichothecene mycotoxins produced, *F. graminearum* can be classified into different chemotypes: chemotype I, which produces DON and/or its acetylated derivatives, and chemotype II, which produces NIV and/or 4-ANIV [14]. The DON chemotype can be further broken down into chemotype IA (producing DON and 3-ADON) and IB (producing DON and 15-ADON [15,16]. Additionally, some *Fusarium* isolates that produce both NIV and DON (NIV/DON chemotype) have been described as “unknown” chemotypes [17]. The most well-known and commonly found mycotoxins produced by different isolates of *F. graminearum* are DON and ZEN, although 3-ADON and 15-ADON can also be found frequently [18,19], accounting for 0%–16% of DON content [9].

Mycotoxin biosynthesis is a complex process that is regulated by genetic mechanisms, which can be affected by various environmental stimuli [20]. It has been reported that both the growth of fungi and their toxigenic potential can be affected by several environmental factors, including temperature, water activity, pH, and nutrient composition and availability [20,21,22,23]. Published data suggest that the same fungus can produce a different range of mycotoxins under different conditions, and even the biosynthesis of mycotoxins for toxin-producing strains is not necessary under certain conditions [24,25]. Merhej et al. [26] demonstrated that the pH regulatory factor Pac1 can regulate expression of the Tri genes, which are associated with trichothecene biosynthesis in *F. graminearum* and suggested that the production of trichothecene was induced only under acidic pH conditions.

A published report concerning the impact of environmental factors and fungicides on the growth and deoxinivalenol production of *F. graminearum* isolates from Argentinian wheat, showed that DON production in the presence of fungicides was influenced by complex interactions between water activity, temperature, fungicide concentration and incubation time [27]. However, there are limited data on the influence of environmental factors and their effects on mycotoxin production in *Fusarium* species. A better understanding of the role of culture conditions, including medium pH, incubation temperature and time, as well as their interactions on the production of DON and ZEN, is required, especially for *F. graminearum* strains. On the other hand, with increased concerns on the potential threat of *Fusarium* mycotoxin to human health, there has been a growing demand for mycotoxin standards to use in the relevant studies. In this way, maximizing the production of targeted mycotoxins may be possible by regulating the incubation conditions of *F. graminearum*. Mycotoxins with acceptable purity could be obtained by separation and purification processes and used as reference materials.

Response surface methodology (RSM), one of the most popular optimization techniques, has been widely used to estimate the relationships between a set of controllable experimental factors and obtained data [28,29]. Based on the model, independent variables can be controlled and optimal responses for the production of prime products can be obtained [30]. This methodology can greatly reduce the number of necessary experiments and provide a set of mathematical equations for theoretical process optimization [31]. Box-Behnken design (BBD), consisting of central and middle points on the edges of the cube circumscribed on the sphere, has been widely used for multivariate optimization of RSM. BBD produces a statistical model with fewer design points than central composite designs (CCD) [32].

This work was conducted to investigate the influence of cultivation conditions on the synthesis of mycotoxins and to maximize the production of DON and ZEN from *F. graminearum* using response surface methodology (RSM). After separation and purification, the isolated mycotoxin samples could have potential use as mycotoxin standards or chemical reagents for routine analysis. 

## 2. Results and Discussion

The most well-known and commonly encountered mycotoxins of *F. graminearum* are ZEN, DON, and its acetylated derivatives (3-ADON, 15-ADON). Based on the reported literature, mycotoxin production can be affected by medium composition and environmental conditions, such as incubation temperature, time, and water activity [11,22,23,33]. In this study, the effect of incubation conditions on DON and ZEN production was evaluated, and the optimization of independent variables was performed by RSM.

### 2.1. Model Fitting and Statistical Analysis

Preliminary experiments showed that all of the variables examined in this study had an effect on DON and ZEN production. Therefore, the effects of three variables, including medium pH, incubation temperature and time on the responses (levels of DON and ZEN) were examined using Box-Behnken design (BBD). The complete design matrix, together with the response values are shown in Table 1. By applying multiple regression analysis, the predicted response *Y* for the yields of DON and ZEN could be obtained by the following second-order polynomial Equations (1) and (2):

*Y*_DON_ = −10314.19 + 2303.58*X*_1_ + 322.65*X*_2_ + 220.03*X*_3_ − 8.87*X*_1_*X*_2_ − 16.88*X*_1_*X*_3_ + 4.72*X*_2_*X*_3_ − 165.15*X*_1_^2^ − 8.66*X*_2_^2^ − 3.73*X*_3_^2^ (1)

*Y*_ZEN_ = 18106.60 − 2969.35*X*_1_ − 260.82*X*_2_ − 1090.25*X*_3_ − 93.59*X*_1_*X*_2_ + 56.38*X*_1_*X*_3_ − 10.59*X*_2_*X*_3_ + 521.77*X*_1_^2^ + 19.47*X*_2_^2^ + 29.44*X*_3_^2^ (2)

where *Y* is the predicted response (levels of DON or ZEN), and *X*_1_, *X*_2_ and *X*_3_ are three independent variables, namely, media pH, incubation temperature, and time. 

The results of the analysis of variance by Fisher’s F test, goodness-of-fit and the adequacy of the models are summarized in Table 2. For DON, a highly significant quadratic regression model was obtained with a high F value and a very low *p*-value (*p* = 0.0001 < 0.01), indicating that the combined effects of all the independent variables contributed significantly to maximizing the response [34]. The lack of fit (*p* = 0.1110 > 0.05) also suggested that the obtained data was a good fit with the model. The value of the determination coefficient (*R*^2^ = 0.9983) implied that the sample variation of 99.83% for DON production was attributable to the independent variables. The adjusted correlation coefficient (*R*^2^_adj_ = 0.9962) and predicted determination coefficient (*R*^2^_pre_ = 0.9793) for Equation (1) were also satisfactory to confirm the significance of the model. The higher of the adjusted correlation coefficients showed a better degree of correlation between the actual and predicted values [35]. 

For Equation (2), the *p*-value (*p* < 0.0001) and *F*-value (*F* = 2884.71) of the model suggested that it was significant. The value of the determination coefficient (*R*^2^ = 0.9997) indicated that approximately 0.03% of the total variance was not explained by the response. The adequacy of the model was also justified by the obtained adjusted determination coefficient (*R*^2^_adj_ = 0.9994) and predicted determination coefficient (*R*^2^_pre_ = 0.9958). The aforementioned data suggested that the mathematical model was reliable and could be used for ZEN production.

The model coefficients obtained by regression analysis for each variable are shown in Table 2. The significance of each variable and the strength of any interaction between each independent variable were tested using the *p*-value. Table 2 shows that the regression coefficients of all linear, quadratic and interaction terms were significant at the 1% level. The corresponding variables became more significant when the *F*-value was larger and the *p*-value was smaller [36]. From the values of the coefficients in the regression model, the order in which the independent variables affected DON production was in line with that of ZEN, which was pH (*X*_1_) > Time (*X*_3_) > Temperature (*X*_2_).

### 2.2. Response Surface Analysis

Using RSM, the effects of the independent variables (medium pH, incubation temperature and time) and their interactions on the yield of DON and ZEN could be graphically described using three-dimensional response surface plots and two-dimensional contour plots. Based on the regression model described in Equations (1) and (2), the responses were predicted and the optimum values for the production of DON and ZEN were determined [37,38].

**Figure 1:**
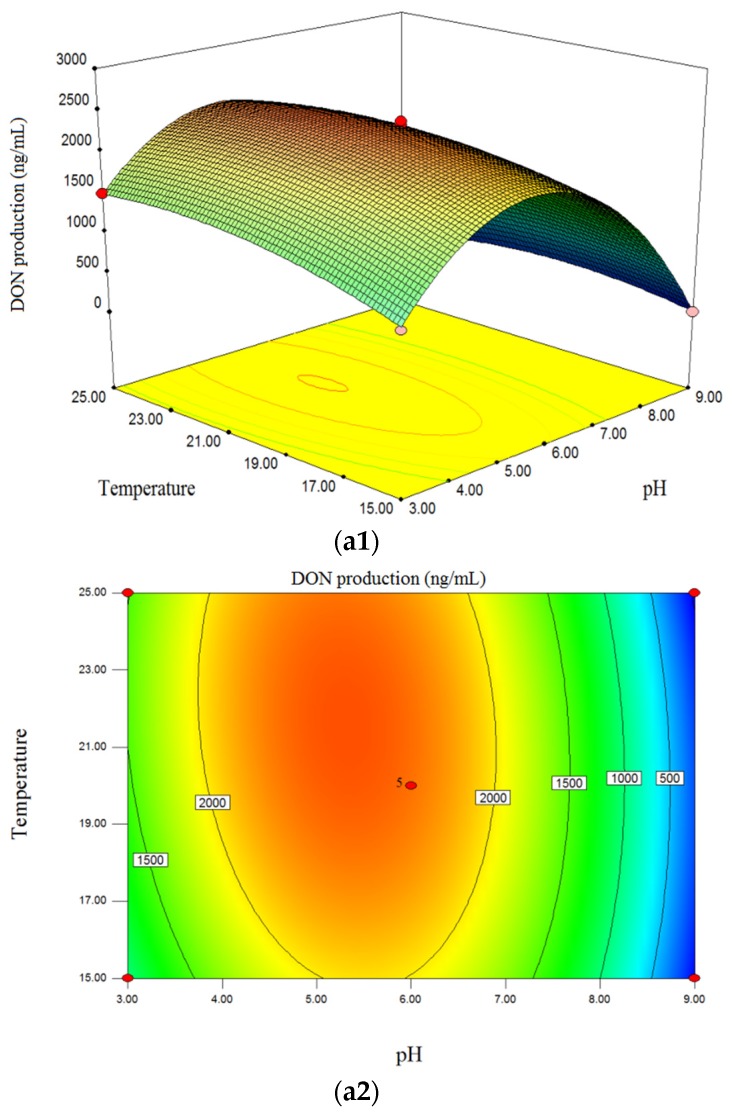

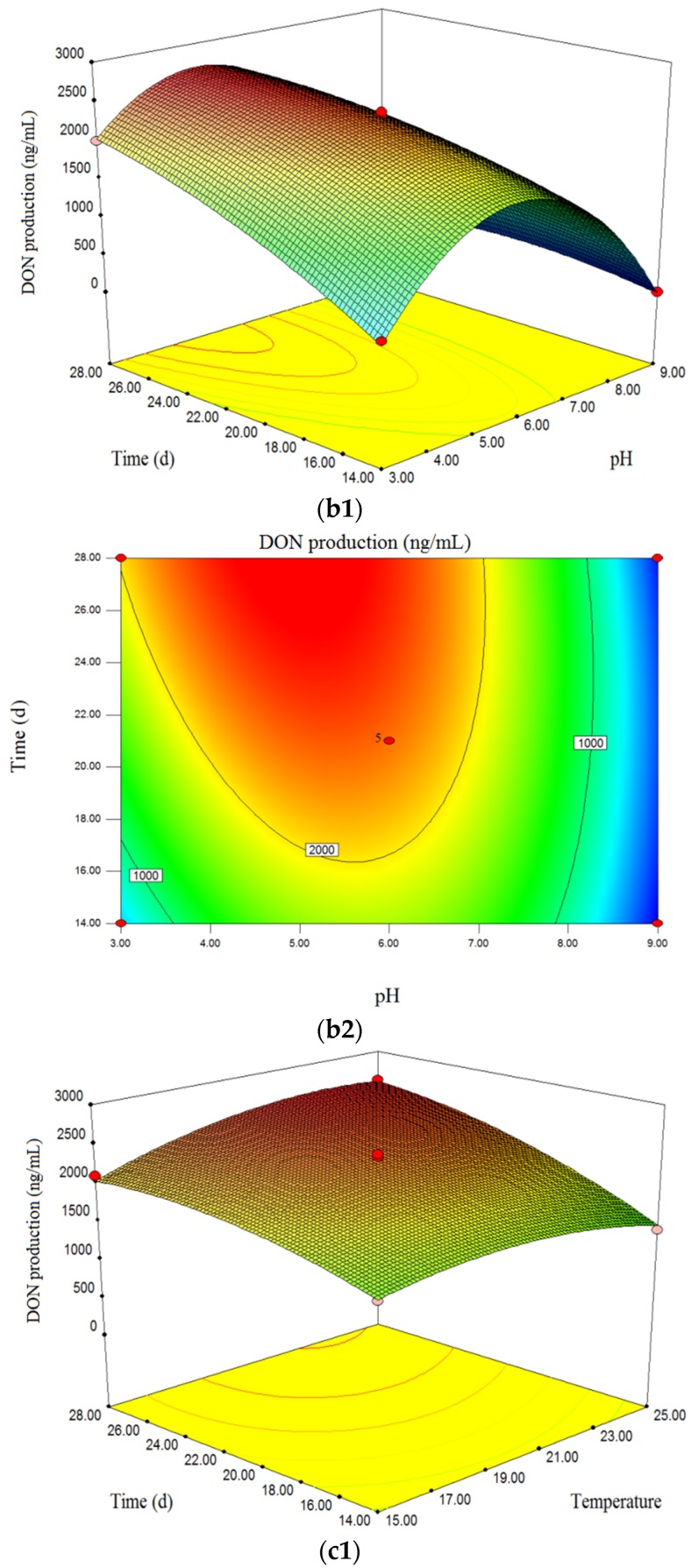

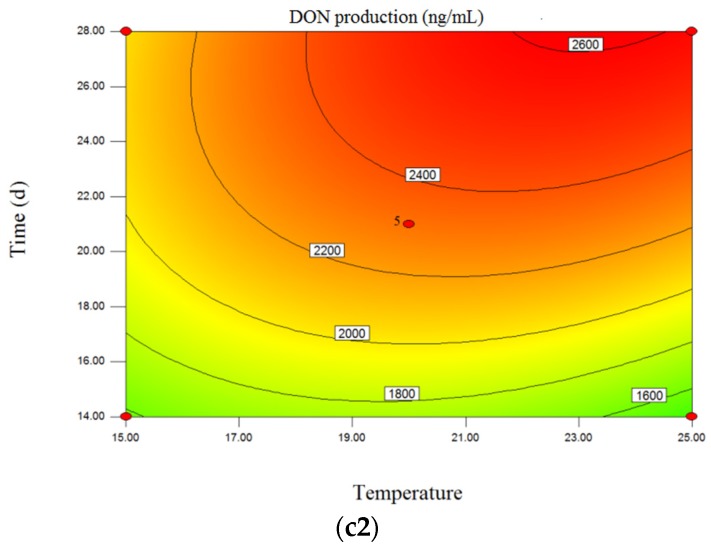
Three-dimensional response surface plots and corresponding contour plots of variables for DON production of *F.graminearum* strain 29. The interactions between medium pH and incubation temperature (**a1**,**a2**), pH and incubation time (**b1**,**b2**), and incubation temperature and time (**c1**,**c2**) are shown.

**Figure 2:**
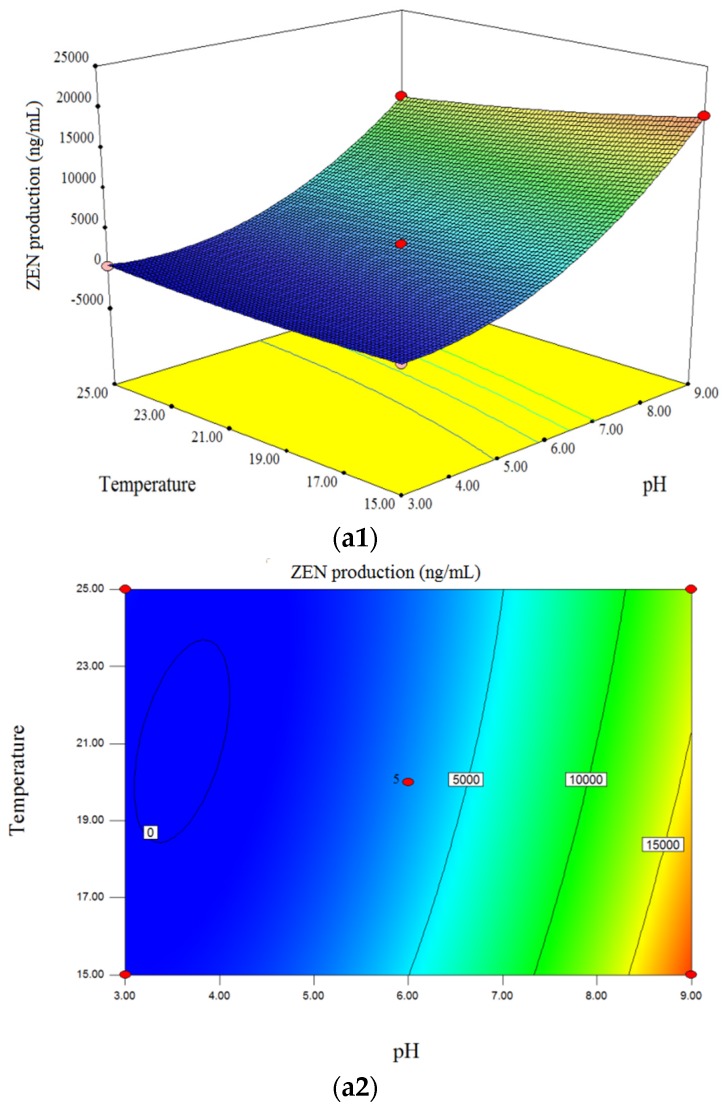

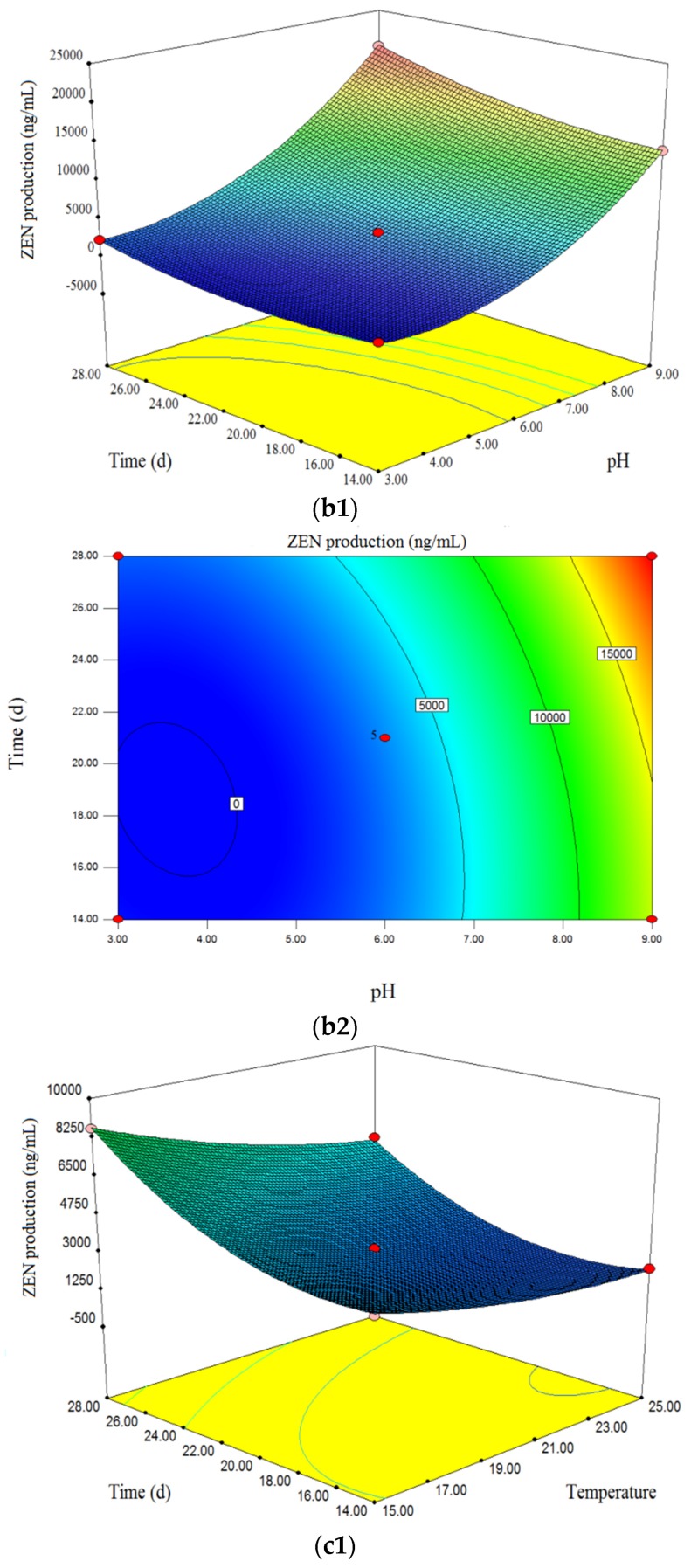

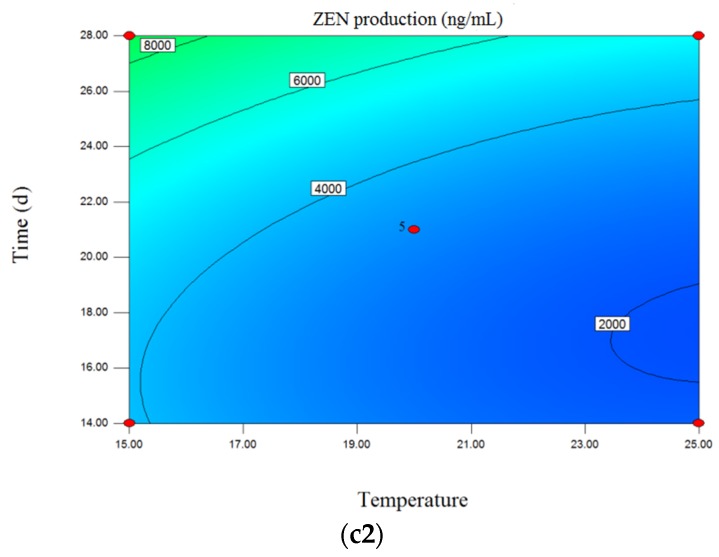
Three-dimensional response surface plots and corresponding contour plots of variables for ZEN production of *F.graminearum* strain 29. The interactions between medium pH and incubation temperature (**a1**,**a2**), pH and incubation time (**b1**,**b2**), and incubation temperature and time (**c1**,**c2**) are shown.

The interaction effect of initial medium pH (*X*_1_) and incubation temperature (*X*_2_) on of DON and ZEN production is shown in Figure 1 (a1,a2) and Figure 2 (a1,a2). The pH value significantly affected the DON production, and was the most important factor which affected the response (Figure 1 (a1,a2)). This showed that DON levels tended to increase with increasing medium pH values within the range of 3.00–4.80, irrespective of the incubation temperature used, whereas DON concentration decreased with increasing pH in the ranged of 5.00–9.00. When pH was set to 9.00 (alkaline environment) irrespective of other factors, the model predicted that no DON would be produced by *F. graminearum*. This was in accordance with published reports, where Merhej et al. showed that the pH regulatory factor Pac1 regulated Tri gene expression and trichothecene production in *F. graminearum*, and the synthesis of trichothecene is induced only under acidic conditions [26]. Additionally, Gardiner et al. suggested that low extracellular pH both promoted and was required for DON production in *F. graminearum* [39]. Although low pH induces DON accumulation, it is not suitable for the growth and development of fungi [40], therefore the optimized pH was 4.70–5.10 in this study. For the effect of temperature, in the range of 15.0 °C–24.0 °C, the contour plot showed an increase in DON production with rising temperature, although DON levels tended to decrease when the temperature exceed 24 °C. Figure 2 (a1,a2) shows that ZEN production increased with increasing pH (4.80–9.00) and reached a maximum level at pH 9.00. However, the yield of ZEN seemed to be reduced with increasing temperature (15.5 °C–25 °C), meaning that temperature negatively affected the production of ZEN when pH and incubation time were fixed. Additionally, there were significant interaction effects between pH and temperature on ZEN production (Table 2, Figure 2 (a1,a2).

Figure 1 (b1,b2) shows that there was an increasing trend of DON level with increasing incubation time (14–28 days) when other factors were fixed. The same trend could be seen from three dimensional (3D) surface plot (Figure 2 (b1,b2)), which indicated that there was positive correlation between ZEN levels and incubation time. Thus, the maximum content of DON and ZEN was obtained when the fungi were cultivated for 28 days. As for DON, based on the interaction between pH and incubation time, the optimized plot was determined at pH of 4.50–5.20 and an incubation time of 28 days. 

The interactions of incubation temperature and time on DON and ZEN levels are represented in Figure 1 (c1,c2) and Figure 2 (c1,c2). As shown in Figure 1c (c1,c2), both an increase in incubation temperature and time within the designed range resulted in increased DON yield, whereas no significant increase was found when the temperature reached 24 °C–25 °C. Thus, the maximum DON level was obtained when the fungi were cultivated for 28 days at 24 °C. On the contrary, temperature negatively affected ZEN production. The optimized condition for ZEN production was cultivation for 28 days at 15 °C. According to Garcia et al., the optimal temperature for mycotoxin production from *F. graminearum* was 15 °C for zearalenone and 20 °C for deoxynivalenol [22]. However, Llorens et al. showed that temperature significantly affected mycotoxin production and the optimal values were 28 °C, 20 °C and 15 °C for DON, NIV and 3-ADON production, respectively [23]. It was found that there were some differences in the optimal temperature for mycotoxin production among published reports. This may be attributed to the studied strains, which were from different localities and countries, and the different compositions of the culture medium. In this study, the optimal temperature for DON production was 23 °C–24 °C and for ZEN production was 15 °C–15.5 °C.

### 2.3. Optimization of Independent Variables and Validation of the Model

In order to obtain the desired response goal, optimization was performed by applying a Box-Behnken design of RSM to predict the optimum levels of the independent variables (medium pH, incubation temperature and time). Numerical and graphical optimization procedures were conducted to find the optimal process. Considering the experimental purpose, two different goals were determined: (1) optimize the conditions to achieve maximum levels of DON and ZEN, separately; and (2) find a combined condition to balance the production of DON and ZEN and obtain a satisfactory total yield for DON and ZEN. Based on the first target, the maximum DON level of 2811.17 ng/mL, under the condition pH 4.91, incubation temperature of 23.75 °C and 28 days of incubation, was predicted. However, the predicted maximum level of ZEN (23789.70 ng/mL) was produced at a pH of 9.0, an incubation temperature of 15.05 °C and incubation time of 28 days.

As the most favorable conditions for DON and ZEN production were inconsistent, we attempted to balance the two levels. The program combined the individual desirability into a single number, and then attempted to maximize this function [38]. The optimum working conditions and respective responses (DON and ZEN) were estimated, and the desirable solutions were found. Under these conditions, the model predicted DON and ZEN levels of 1718.74 ng/mL and 11797.9 ng/mL, respectively. The optimum conditions were pH of 6.86, incubation temperature of 17.75 °C, and an incubation time of 28 days.

To verify the accuracy of the model and equations, an optimization experiment was conducted under the following conditions: (i) pH value of 6.80, incubation temperature of 17.7 °C, and incubation time of 28 days; (ii) pH value of 4.90, incubation temperature of 23.80 °C, and incubation time of 28 days; and (iii) pH value of 9.00, incubation temperature of 15.00 °C, and incubation time of 28 days. The obtained DON and ZEN levels were 1658.05 ng/mL and 118103.40 ng/mL, respectively, under condition (i), while 2771.35 ng/mL of DON was obtained under condition (ii), and 23218.88 ng/mL of ZEN was obtained under condition (iii). These data showed that the actual values were all close to the predicted results. This suggested that the optimum conditions determined by RSM could be used to optimize the culture conditions for DON and ZEN production.

Several reports have shown that acid and alkaline environments can markedly affect the production of trichothecene mycotoxins, especially for DON, and suggested that DON could be only produced under acidic conditions from *F. graminearum* [23,36,37]. However, few publications are available concerning the acid-base conditions required for ZEN production. Our data showed that a higher pH value (weakly alkaline) was favorable for ZEN accumulation. This knowledge can provide further information to control and prevent mycotoxin contamination in the planting, harvesting and storage of cereals. 

### 2.4. Purification and Purity of Mycotoxin Samples for Potential Analytical Standards

Based on our early experiments, we obtained acceptable recoveries (>80%) of mycotoxins, including DON and ZEN, following solvent extraction and hexane partitioning (data not shown). According to Wu et al., approximately 80% of fusaproliferin from cultures of *Fusarium subglutinans* was recovered after hexane partitioning. This demonstrated that hexane partitioning was an effective way to remove impurities from crude fusaproliferin extracts prior to HPLC [41].

**Figure 3:**
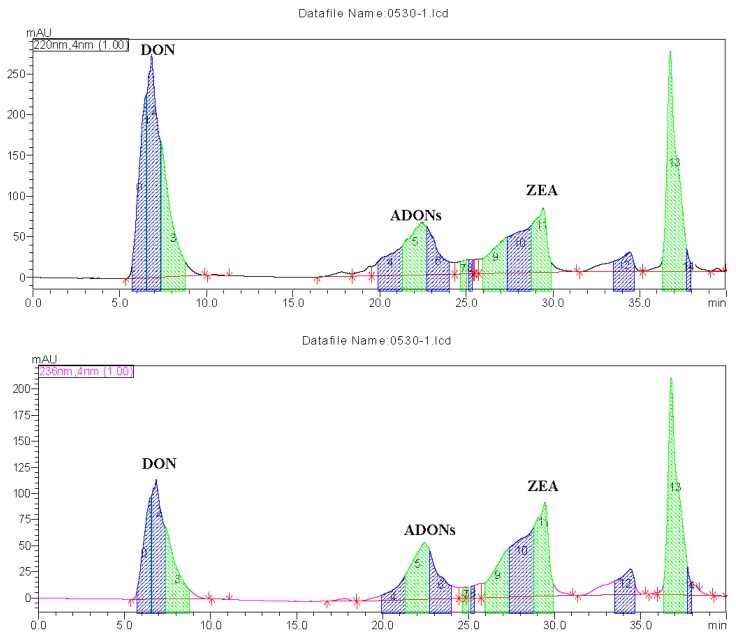
UV dual-wavelength spectrophotometry chromatogram of mycotoxins analyzed by preparative HPLC.

After extraction of mycotoxins from potato dextrose broth (PDB) medium, DON and ZEN were isolated and purified by preparative HPLC. As shown in Figure 3, the targeted fractions were collected by an automatic fraction collector. The collected fractions were concentrated using a rotary evaporator and a freeze–dryer to a dry powder. Moisture content was then determined by UPLC-MS/MS and analytical HPLC based on comparing their retention times and MS data with the analytical standards and published data [42]. DON and ZEN purity was measured by areas of peak normalization method of HPLC [43], which showed that DON was >91% pure, while ZEN was >87% pure, suggesting that these isolated mycotoxins could potentially be used as analytical standards. In this study, a common nutrient broth of PDB with simple constituents was used. However, the yield of DON and ZEN might have been lower than some other *F. graminearum* isolates that were incubated with cereals, such as wheat and maize. Molto et al. showed that twenty 27 of 27 *F. graminearum* isolates produced deoxynivalenol (384–5745 µg/kg) and 13/27 produced zearalenone (200–35,045 µg/kg) when they were cultured with Argentinian maize [44]. Due to the simple purification steps, desirable yields, and acceptable purity of the isolated mycotoxins, their production is feasible, and the purified mycotoxin samples may be used as potential analytical standards or chemical reagents for routine analysis.

## 3. Conclusions

The proposed model equations illustrated both the quantitative effect and the interactions of the variables on mycotoxin production. By applying RSM, the optimized conditions for *F. graminearum* strain 29 were determined and desirable yields of the targeted mycotoxins were obtained, which could be further supplied as mycotoxin standards after purification and separation. Under the optimized conditions, maximal levels of DON (2811.17 ng/mL) and ZEN (23789.70 ng/mL) were obtained. A combined solution with a desirable total yield of both DON and ZEN was obtained, using a medium pH of 6.86, incubation temperature of 17.76 °C, and time of 28 days. The corresponding experimental values, from the validation experiments, fitted well with these predictions, suggesting that RSM could be applied to optimize *Fusarium* mycotoxin production. After extraction, separation and purification processes, the isolated mycotoxins were obtained with a desirable yield and acceptable purity. The mycotoxins could be used as potential analytical standards or chemical reagents for routine analysis.

This work also shows that the ambient pH value is a crucial factor for *F. graminearum* toxin production. Acidic pH is a determinant for DON production, while an alkaline environment is favorable for ZEN accumulation. As for incubation temperature, higher temperatures (23–25 °C) are suitable for DON accumulation, although a lower temperature (approximately 15 °C) favors a higher yield of ZEN.

## 4. Materials and Methods

### 4.1. Chemicals and Reagents

Mycotoxin standards, including DON and ZEN were purchased from Sigma-Aldrich (Sigma-Aldrich, St. Louis, MO, USA). Mycotoxin solid standards were dissolved in methanol (100 μg/mL) and stored at −20 °C in a sealed vial until use. All organic solvents, including methanol, acetonitrile and formic acid were of HPLC grade and were supplied by Fisher Scientific (Shanghai, China). Sodium hydroxide and concentrated hydrochloric acid were all analytical reagents and purchased from Beijing Chemical Reagent (Beijing, China). Pure water was obtained from a milli-Q system (Millipore, Billerica, MA, USA).

### 4.2. Fungal and Inoculum Preparation

Three fungal strains were kindly supplied by Professor Xiaoming Wang, Institute of Crop Science, Chinese Academy of Agricultural Sciences (CAAS, Beijing, China). Strains 001, 029 and 075 were obtained by single spore isolation from diseased corn spikes in China, which originated in China. The identification of the *F. graminearum* isolates was confirmed at the molecular level by a multiplex-PCR with primers Tri7F340/Tri7R965, 3551H/4056H, Tri3F971/Tri3R1679 and Tri3F1325/Tri3R1679 [45]. All strains belonged to the *F. graminearum* DON chemotype and can be further defined as the 15-ADON chemotype, which mainly produce ZEN, DON, and some of its acetyl-derivatives, namely, 15-ADON and 3-ADON. These isolates were incubated for 4–5 days at 25 °C on potato dextrose agar (PDA; potato extract, 4.0 g/L; dextrose, 20.0 g/L; agar, 15.0 g/L). Colonies were subcultured on new PDA plates and stored on PDA slants at 4 °C for further use. Following preliminary experiment to compare toxin production in the strains, strain 029 was chosen due to its high level of toxin production. Plugs (3 mm diameter) were collected with a cork borer from the margin of colonies grown on PDA medium in 9 cm Petri dishes for seven days at 25 °C. All the plugs were prepared and incubated in the fluid nutrient medium of potato dextrose broth (PDB).

### 4.3. Culture Conditions for Mycotoxin Production of F.graminearum Strain 29

Potato dextrose broth was used (PDB; potato extracts, 6.0 g/L; dextrose, 20.0 g/L) as the culture medium for fungal growth and mycotoxin production. The fungi was incubated in 200 mL of PDB for each run with different environmental factors. Medium pH (3–9), incubation temperature (15–25 °C), and time (14–28 days) were evaluated to find the most suitable culture condition for mycotoxin accumulation. 

### 4.4. Experimental Design for Mycotoxin Production of F. graminearum Strain 29

To optimize the toxin-producing conditions for *F. graminearum*, the different culture conditions, including medium pH, incubation temperature and time, were evaluated using RSM. RSM was performed to assess the relationship between responses (mycotoxin production) and three independent variables, as well as to optimize the relevant conditions of variables aimed to predict the best value of responses [38]. 

A three-factor, three-level design was developed by Box-Behnken design (BBD; Design Expert software, Trial Version 8.0.6, Stat-Ease Inc., Minneapolis, MN, USA) to obtain a second-order polynomial model. Thereby, a suitable combination of variables could be determined to give the highest production of the targeted mycotoxins. Three variables, namely, medium pH (*X*_1_), incubation temperature (*X*_2_), and time (*X*_3_) and their appropriate ranges were determined based on single-factor experiments.

The coded and actual levels of the independent variables are shown in Table 3. A total of 17 experimental runs were carried out, and five replicates at the center point of the design were used for estimating the experimental error sum of squares. Triplicate analyses were performed at all design points in a random order.

The experimental data was analyzed using multiple regression and the second order polynomial model fitted for predicting optimal levels is expressed in Equation (3):

(3) $$Y=\beta_{0}+\beta_{1}X_{1}+\beta_{2}X_{2}+\beta_{3}X_{3}+\beta_{12}X_{1}X_{2}+\beta_{13}X_{1}X_{3}+\beta_{23}X_{2}X_{3}+\beta_{11}X_{1}^{2}+\beta_{22}X_{2}^{2}+\beta_{33}X_{3}^{2}$$

where *Y* is the predicted response; *X*_1_, *X*_2_, and *X*_3_ are independent variables; β_0_ is a constant coefficient; β_1_, β_2_, β_3_ are linear regression coefficients; β_12_, β_13_, β_23_ are interactive regression coefficients; and β_11_, β_22_, β_33_ are quadratic regression coefficients.

### 4.5. Mycotoxin Determination

UPLC–MS/MS was used for the analysis and determination of DON and ZEN. The methods for extraction, clean-up and determination of DON and ZEN from the cultures was based on previously published work [9] with slight modifications. Briefly, 5 mL culture filtrate was extracted with 20 mL of acetonitrile for 30 min using an automatic shaker. After centrifugation, 8 mL of the supernatant was passed through a MycoSep 226 Aflazon + multifunctional column (Romer Labs, Inc. Union, MO, USA) and 4 mL of the purified extract was evaporated to dryness under a stream of nitrogen. The residue was then dissolved in 1 mL of methanol–water (50/50, *v*/*v*), followed by filtering, and was subsequently used for analysis. Data acquisition and processing were performed using MassLynx v4.1 and Quanlynx (Waters, Milford, MA, USA).

### 4.6. Mycotoxins Extraction, Purification and Analysis for Potential Analyte Standards

#### 4.6.1. Mycotoxin Extraction from Growth Medium

The extraction of DON and ZEN was carried out after 28 days of incubation in PDB based on the optimized culture conditions. The cultures were filtered with filter paper, and the mycelia were extracted with methanol, then the filtrate and extract were combined and concentrated under reduced pressure using a rotary evaporator at 65 °C. Subsequently, methanol/water (80/20, *v*/*v*) was added and the extracts were washed with hexane (60 mL) to defat. The aqueous and methanol layers were collected, concentated and filtered through a 0.45 µm nylon filter, and stored in brown glass vials at −20 °C until further purification and isolation [46,47].

#### 4.6.2. Mycotoxin Seperation and Purification by Preparative Column HPLC

The mycotoxin-containing extracts dissolved in methanol were purified by preparative HPLC. The LC system was equipped with a LC-20APpump, SIL-10AP injector, FRC-10A fraction collector and a diode array detector (DAD) (Shimadzu, Kyoto, Japan), and a reverse-phase SHIM-pack PRC-ODS C18 column (20 mm × 250 mm, 5 μm, Shimadzu, Kyoto, Japan ) was used for mycotoxin seperation. The mobile phases were acetonitrile/methanol (50:50, *v*/*v*, solvent A) and H_2_O (solvent B) at a flow rate of 8 mL/min. The detector was set at λ_1_ = 220 nm for DON and its acetylated derivatives, and λ_2_ = 236 nm for ZEN. The linear gradient program started from 20% A for 8 min and increased to 80% A within 1 min, then held for 11 min. Finally, the initial composition of 20% A was re-established followed by equilibration for 5 min. LC retention times and UV absorbance profiles of the purified mycotoxins were compared to those of standard solutions. The targeted fractions were collected by a fraction collector. The LC-purified mycotoxin fractions were concentrated using a rotary evaporator at 45 °C, frozen overnight at −20 °C and then dried in a freeze–dryer for 48 h.

#### 4.6.3. Identification and Purity of the Isolated Mycotoxin Samples

The identfication of the isloated mycotoxins was performed by UPLC-MS/MS with ESI (+) mode and analytical HPLC equipped with a DAD detector by comparing their retention times and MS data with the analytical standards and published data [9,42]. As for MS detection, the precursor ion (*m*/*z*) of 297.28, quantitative ion (Q) of 249.18 and qualitative ion (q) of 203.16 were used for DON analysis, while the precursor ion (*m*/*z*) of 319.05, quantitative ion (Q) of 283.03 and qualitative ion (q) of 187.36 were used for ZEN analysis. The purity of the isolated samples was determined by analytical HPLC using areas of peak normalization method [43].

### 4.7. Statistical Analysis

Stat-Ease software (Design-Expert 8.0.5 version, Stat-Ease Corporation, Minneapolis, MN, USA) was used for the regression analysis of the data, and to plot the response surface graphs. Analysis of variance (ANOVA) was performed and the values were considered significant when *p* < 0.05. The variability and accuracy of the model were determined according to the regression coefficient (*R*^2^) and lack of fit, respectively. The response surface and contour plots of the predicted responses of the model were used to assess interactions between the significant factors. Additionally, numerical optimization was carried out by performing three-dimensional response surface analysis of the independent and dependent variables.

## Figures and Tables

**Table 1:** Experimental design used in the response surface methodology studies based on three independent variables and the observed responses of *F. graminearum* strain 29.

No.	*X*_1_	*X*_2_	*X*_3_	Mycotoxin Levles (ng/mL)	
	PH	Temperature (°C)	Time (day)	DON	ZEA
1	−1 (3)	−1 (15)	0 (21)	961.02 ± 35.78	419.97 ± 30.25
2	1 (9)	−1 (15)	0 (21)	<LOD	19084.20 ± 1102.56
3	−1 (3)	1 (25)	0 (21)	1493.05 ± 91.23	609.66 ± 41.35
4	1 (9)	1 (25)	0 (21)	<LOD	13457.90 ± 978.25
5	−1 (3)	0 (20)	−1 (14)	585.59 ± 39.75	818.34 ± 54.25
6	1 (9)	0 (20)	−1 (14)	<LOD	13941.4 ± 886.14
7	−1 (3)	0 (20)	1 (28)	2003.54 ± 123.47	2288.42 ± 231.52
8	1 (9)	0 (20)	1 (28)	<LOD	20047.30 ± 1203.59
9	0 (6)	−1 (15)	−1 (14)	1559.09 ± 101.27	4118.31 ± 334.59
10	0 (6)	1 (25)	−1 (14)	1405.5 ± 89.58	2259.09 ± 189.67
11	0 (6)	−1 (15)	1 (28)	2098.03 ± 128.71	8661.47 ± 678.15
12	0 (6)	1 (25)	1 (28)	2605.26 ± 134.55	5319.75 ± 408.14
13	0 (6)	0 (20)	0 (21)	2276.33 ± 121.30	3198.34 ± 278.27
14	0 (6)	0 (20)	0 (21)	2313.33 ± 108.24	3133.31 ± 256.77
15	0 (6)	0 (20)	0 (21)	2332.08 ± 107.57	3126.45 ± 281.73
16	0 (6)	0 (20)	0 (21)	2283.66 ± 119.35	3156.87 ± 312.25
17	0 (6)	0 (20)	0 (21)	2376.34 ± 123.83	3185.81 ± 243.51

**Table 2:** ANOVA for response surface quadratic models for targeted mycotoxin production.

Source	Sum of Squares	df	Mean Squares	*F*-Value	*p*-Value
**DON**					
Model	1.520 × 10^7^	9	1.689 × 10^6^	461.94	0.0001
*X*_1_	3.179 × 10^6^	1	3.179 × 10^6^	869.41	<0.0001
*X*_2_	98051.42	1	98051.42	26.82	0.0013
*X*_3_	1.246 × 10^6^	1	1.246 × 10^6^	340.77	0.0001
*X*_1_ *X*_2_	70763.98	1	70763.98	19.35	0.0032
*X*_1_ *X*_3_	5.030 × 10^5^	1	5.030 × 10^5^	137.57	<0.0001
*X*_2_ *X*_3_	1.092 × 10^5^	1	1.092 × 10^5^	29.86	0.0009
*X*_1_^2^	9.302 × 10^6^	1	9.302 × 10^6^	2544.13	<0.0001
*X*_2_^2^	1.974 × 10^5^	1	1.974 × 10^5^	53.99	0.0002
*X*_3_^2^	1.408 × 10^5^	1	1.408 × 10^5^	38.51	0.0005
Residual	25592.79	7	3656.11		
Lack of Fit	19068.00	3	6356.00	3.90	0.1110
Pure Error	6524.79	4	1631.20		
Cor Total	1.523 × 10^7^	16			
**ZEN**					
Model	6.534 × 10^8^	9	7.260 × 10^7^	2884.71	<0.0001
*X*_1_	4.882 × 10^8^	1	4.882 × 10^8^	19398.73	0.0001
*X*_2_	1.414 × 10^7^	1	1.414 × 10^7^	562.05	0.0001
*X*_3_	2.918 × 10^7^	1	2.918 × 10^7^	1159.65	0.0001
*X*_1_ *X*_2_	7.885 × 10^6^	1	7.885 × 10^6^	313.31	0.0001
*X*_1_ *X*_3_	5.607 × 10^6^	1	5.607 × 10^6^	222.80	0.0001
*X*_2_ *X*_3_	5.495 × 10^5^	1	5.495 × 10^5^	21.83	0.0023
*X*_1_^2^	9.285 × 10^7^	1	9.285 × 10^7^	3689.54	0.0001
*X*_2_^2^	9.977 × 10^5^	1	9.977 × 10^5^	39.65	0.0004
*X*_3_^2^	8.764 × 10^6^	1	8.764 × 10^6^	348.24	0.0001
Residual	1.762 × 10^5^	7	25166.28		
Lack of Fit	175500	3	57393.40	5.76	0.0710
Pure Error	3983.75	4	995.94		
Cor Total	6.536 × 10^8^	16			

**Table 3:** Experimental range and levels of the independent variables.

Variables	Symbols	Range and Levels		
		Low (−1)	Medium (0)	High (+1)
Temperature (°C)	*X*_1_	15	20	25
pH	*X*_2_	3	6	9
Time (d)	*X*_3_	14	21	28
